# Distribution of *pfmdr1* polymorphisms in *Plasmodium falciparum* isolated from Southern Thailand

**DOI:** 10.1186/1475-2875-13-117

**Published:** 2014-03-27

**Authors:** Mathirut Mungthin, Somchai Intanakom, Nantana Suwandittakul, Preeyaporn Suida, Sakultip Amsakul, Naruemon Sitthichot, Suwich Thammapalo, Saovanee Leelayoova

**Affiliations:** 1 Department of Parasitology, Phramongkutklao College of Medicine, Ratchawithi Rd, Bangkok 10400, Thailand; 2 Office of Disease Prevention and Control 12, Department of Disease Control, Ministry of Public Health, Songkhla 90000, Thailand; 3 Vecter Born Disease Control Center 12.1, Ministry of Public Health, Yala 95000, Thailand; 4 Office of Disease Prevention and Control 11, Ministry of Public Health, Nakonsitammarat 80000, Thailand

## Abstract

**Background:**

Drug resistance in *Plasmodium falciparum* is a major problem in malaria control especially along the Thai-Myanmar and Thai-Cambodia borders. To date, a few molecular markers have been identified for anti-malarial resistance in *P. falciparum,* including the *P. falciparum* chloroquine resistance transporter (*pfcrt*) and the *P. falciparum* multidrug resistance 1 (*pfmdr1*). However no information is available regarding the distribution pattern of these gene polymorphisms in the parasites from the Thai-Malaysia border. This study was conducted to compare the distribution pattern of the *pfcrt* and *pfmdr1* polymorphisms in the parasites from the lower southern provinces, Thai-Malaysia border and the upper southern provinces, Thai-Myanmar border. In addition, *in vitro* sensitivities of anti-malarial drugs including chloroquine, mefloquine, quinine, and artesunate were determined.

**Methods:**

In all, 492 *P. falciparum*-positive blood samples were collected from the lower southern provinces: Songkhla, Yala and Narathiwas. From the upper southern part of Thailand, Ranong and Chumphon, 66 samples were also collected. Polymorphisms of the *pfcrt* and the *pfmdr1* gene were determined using PCR techniques. *In vitro* sensitivities of anti-malarial drugs were determined using radioisotopic method.

**Results:**

All parasites from both areas contained the *pfcrt* 76 T allele. The *pfmdr*1 86Y allele was significantly more common in the parasites isolated from the lower southern areas. In contrast, the *pfmdr1* 184F allele was predominant among the parasites from the upper southern areas especially Ranong. In addition, the parasites from Ranong contained higher copy numbers than the parasites from other provinces. All adapted parasite isolates exhibited CQ-resistant phenotype. Neither QN nor MQ resistance was detected in these isolates.

**Conclusion:**

The parasites from Thai-Malaysia border exhibited different resistant patterns compared to other areas along the international border of Thailand. This information will be useful for anti-malarial drug policy in Thailand.

## Background

Multidrug resistance in *Plasmodium falciparum* has been a major problem in malaria control along the international borders of Thailand especially, Thai-Myanmar and Thai-Cambodia border [1]. Artemisinin-based combination therapy (ACT), using a combination of artesunate (ART) and mefloquine (MQ), has been introduced for the treatment of uncomplicated falciparum malaria to address this problem [2]. In the past few years, emergence of artemisinin resistance in these areas is a matter of concern [3,4]. Prolonged parasite clearance has now been used as the indicator of artemisinin resistance [4].

A few molecular markers have been identified for anti-malarial resistance in *P. falciparum*. The *P. falciparum* chloroquine resistance transporter (*pfcrt*) has been identified as the main determinant of chloroquine (CQ) resistance [5]. A point mutation on the *pfcrt* gene resulting in replacement of lysine by threonine in the PfCRT at codon 76 has been linked to CQ resistance in parasite isolates collected worldwide [6]. The *P. falciparum* multidrug resistance 1 (*pfmdr1*), a gene on chromosome 5 encoding a P-glycoprotein homologue 1 (*pgh1*) also contributes to CQ resistance [7-10]. Several studies have shown that single nucleotide polymorphisms and amplification of the *pfmdr1* gene is also associated with *in vitro* response and clinical efficacy of MQ, an arylaminoalcohol [11-14]. Evidence suggests that the *pfmdr1* gene plays a role in the *in vitro* response to other quinolines such as quinine (QN) and lumefantrine (LF) and artemisinin derivatives [15-18].

To date, the distribution of the *pfcrt* and *pfmdr1* polymorphisms were only reported in the parasites collected from the Thai-Myanmar and Thai-Cambodia borders, but not the Thai-Malaysia border [10,13,14,17,18]. Since different patterns of *pfcrt* and *pfmdr1* polymorphisms in *P. falciparum* exhibit varied anti-malarial drug susceptibilities [10,17,18], knowing the distribution of these polymorphisms in these areas would be useful. In this study, the *pfcrt* and *pfmdr1* polymorphisms in *P. falciparum* isolated from the Thai-Malaysia border were determined compared with the parasites isolated from upper southern provinces, Thai-Myanmar border. In addition, the *in vitro* sensitivities of anti-malarial drugs including CQ, MQ, QN and ART were determined in recently adapted *P. falciparum* isolated from this area.

## Methods

### Blood sample collection

Finger-prick blood (approximately 200 μl) samples from patients who visited malaria clinics of the Office of Disease Prevention and Control 12 (the lower southern provinces including Songkhla, Yala and Narathiwas) and the Office of Disease Prevention and Control 11 (the upper southern provinces including Ranong and Chumphon) were collected onto Whatman No 3 filter paper. At these clinics, Giemsa-stained thick film was performed for the diagnosis of malaria. The dried filter paper samples were kept in plastic zipper bags and sent to the Department of Parasitology, Phramongkutklao College of Medicine, for molecular analysis. A total of 492 microscopically positive *P. falciparum* blood samples were collected from three lower southern provinces in 2009. Of these 492 samples, 43, 215 and 234 samples were collected from Songkhla, Yala and Narathiwas, respectively. Sixty-six samples were also collected from the upper southern part of Thailand, i e, 42 samples from Ranong and 24 samples from Chumphon. All these samples were confirmed to be *P. falciparum* positive by PCR technique as described by Snounou *et al*. [19]. The research protocol was reviewed and approved by the Ethics Committee of the Royal Thai Army Medical Department.

### *Plasmodium falciparum* cultivation and *in vitro* sensitivity assays

Fifteen isolates of *P. falciparum* collected from Yala, a province along the Thai-Malaysia border in 2010 were adapted. Parasites were maintained in continuous cultures using a modification of the method of Trager and Jensen [20]. These isolates were tested against commonly used anti-malarial drugs including CQ, MQ, QN and ART using radioisotopic assay as previously described [21]. Drug IC_50_ (i e concentration of a drug which inhibits parasite growth by 50%) was determined from the log dose/response relationship as fitted by GRAFIT (Erithacus Software, Kent, UK). Each *in vitro* sensitivity experiment was carried out in triplicate. The IC_50_ of each isolate was the mean IC_50_ of at least three independent experiments.

### Genotypic characterization for *pfcrt* and *pfmdr1* genes

Parasite DNA was extracted using the Chelex-resin method [22]. Five μl of DNA preparation was used for a 25 μl PCR reaction. PCR and allele-specific restriction analysis were performed for the detection of the *pfcrt* mutations encoded amino acids at position 76 [23]. Mutations in the *pfmdr1* gene were determined by the nested PCR and restriction endonuclease digestion method developed by Duraisingh *et al*. [15] for detection of the mutations at codons 86, 184, 1034, 1042 and 1246 [24]. K1, a laboratory strain containing the 86Y allele was used as a control for the detection of the N86Y mutation. For the positive control of the 184 F, 1034C, 1042D and 1246Y alleles, 7G8 strain was used. The results with a combined band pattern of undigested and digested fragments were considered mixed alleles. The *pfmdr1* gene copy number was determined by TaqMan real-time PCR (ABI sequence detector 7000; Applied Biosystems) as developed by Price *et al*. [25]. The K1 and DD2 clone containing 1 and 4 *pfmdr1* copies, respectively, was used as the reference DNA sample. The *pfmdr1* and *β-tubulin* amplification reactions were run in duplicate. Relative copy number of the *pfmdr*1 gene was determined as previously described [25].

### Statistical analysis

Data were analysed by STATA/MP, Version 12. Differences of the mean copy number of the *pfmdr1* gene among the parasites from different areas were analysed by Independent *t* test and One-way ANOVA. Post hoc test (Scheffe) for multiple comparisons was used to test for differences among groups. Association between genotypes and *P. falciparum* from different areas was analysed by Chi square test. The level of significance was set at a *p* value of < 0.05.

## Results

### Characterization of the *pfcrt* and *pfmdr1* genes

**Figure 1:**
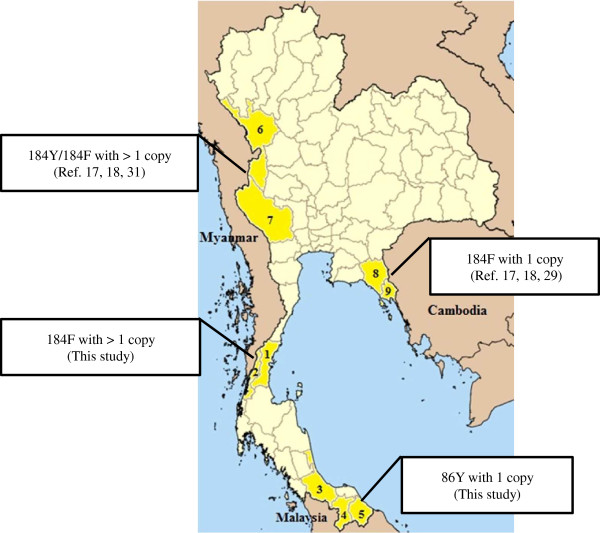
**Predominant *pfmdr1 *genotypes in different areas of Thailand.** The present study locations including two provinces in the upper south, (1) Chumphon and (2) Ranong and three provinces in the lower south, (3) Songkhla, (4) Yala and (5) Narathiwas and previously reported areas including (6) Tak and (7) Kanchanaburi [17,18,31], (8) Chantaburi and (9) Trat [17,18,29].

Genotypic characterization of the parasite isolates from upper and lower southern Thailand is shown in Table 1 and Figure 1. All parasites from both areas contained the *pfcrt* 76T allele. Of the 492 parasite isolates from lower southern Thailand, 474 (96.3%), 16 (3.3%), and two (0.4%) isolates contained the *pfmdr*1 86Y, 184F and 1034C mutations, respectively. The distribution of the *pfmdr*1 polymorphisms of the parasites isolated among three lower southern provinces, i e Yala, Narathiwas and Songkhla, was similar. The *pfmdr*1 86Y allele was significantly more common in the parasites isolated from lower southern areas than those isolated from upper southern areas (Chi square, *p* < 0.001). In contrast, the *pfmdr1* 184F allele was more common in the parasites from upper southern areas (Chi square, *p* < 0.001). In this area, the *pfmdr*1 184F allele was significantly more common in the parasites isolated from Ranong compared to those from Chumphon (Chi square, *p* < 0.001). In contrast, the *pfmdr1* 86Y allele was significantly predominant in the parasites from Chumphon (Chi square, *p* < 0.001). Parasites containing mixed alleles of the *pfmdr1* gene were not detected in all samples.

**Table 1:** **Distribution of the *pfcrt *** **and *pfmdr*** **1 polymorphisms of the parasites isolated from upper and lower southern areas**

**Area**	**No. of isolates**	***pfcrt *** **76T**	**Mean *pfmdr*** **1 copy number**	***pfmdr*** **1 mutations**				
				**86Y**	**184F**	**1034C**	**1042D**	**1246Y**
Upper southern	66	66 (100%)	2.3 ± 1.0*	24 (36.4%)**	42 (63.6%)**	0 (0%)	0 (0%)	0 (0%)
Ranong	42	42 (100%)	2.6 ± 0.8	6 (14.3%)	36 (85.7%)	0 (0%)	0 (0%)	0 (0%)
Chumphon	24	24 (100%)	1.7 ± 0.9	18 (75.0%)	6 (25.0%)	0 (0%)	0 (0%)	0 (0%)
Lower southern	492	492 (100%)	1.2 ± 0.4	474 (96.3%)	16 (3.3%)	2 (0.4%)	0 (0%)	0 (0%)
Yala	215	215 (100%)	1.3 ± 0.5	204 (94.9%)	10 (4.7%)	2 (0.9%)	0 (0%)	0 (0%)
Narathiwas	234	234 (100%)	1.2 ± 0.4	228 (97.4%)	5 (2.1%)	0 (0%)	0 (0%)	0 (0%)
Songkhla	43	43 (100%)	1.2 ± 0.4	42 (97.7%)	1 (2.3%)	0 (0%)	0 (0%)	0 (0%)

*Significant difference between parasites isolated from upper and lower southern area determined by Independent *t* test (*p* < 0.001).

**Significant difference between parasites isolated upper and lower southern area determined by Chi square test (*p* < 0.001).

Determination of the *pfmdr1* gene copy number showed that the isolates from upper southern areas contained the *pfmdr1* copy numbers with the mean of 2.3 (range 1 to 4) which was significantly higher than those found in the parasite from lower southern areas (mean = 1.2, range 1 to 3). Significant differences of the *pfmdr1* copy numbers were found among the parasites from different provinces (One-way ANOVA, *p* < 0.001). Multiple comparison indicated that the parasites from Ranong contain more copy number than the parasites from Chumphon, Yala, Narathiwas and Songkhla (all *p* < 0.001). The parasites from Chumphon also had more copy number than the parasites from lower southern areas, i e Yala, Narathiwas and Songkhla (*p* = 0.007, *p* = 0.004, and *p* = 0.012, respectively).

### *In vitro* anti-malarial sensitivities against 15 *Plasmodium falciparum* isolates from Yala

Table 2 shows *in vitro* response of parasites isolated from Yala to four commonly used anti-malarial drugs. All isolates exhibited CQ-resistant phenotype [26]. Neither QN resistance (QN IC50 of > 800 nM) [27] nor MQ resistance (MQ IC_50_ of > 30 nM) [28] was detected in these isolates. All isolates contained the *pfcrt* 76T and a single copy of the *pfmdr1* gene with the 86Y allele.

**Table 2:** ***In vitro *** **anti-malarial sensitivities in 15 *P. falciparum *** **isolates from Yala**

	**Min IC**_**50 **_**(nM)**	**Min IC**_**50 **_**(nM)**	**Mean IC**_**50 **_**(nM) ± SD**
Chloroquine	63.0	189.7	129.2 ± 45.2
Quinine	102.7	278.2	185.2 ± 61.7
Mefloquine	10.7	24.5	16.6 ± 3.9
Artesunate	3.0	4.4	3.8 ± 0.5

## Discussion

Majority of *P. falciparum* isolates collected from three provinces along the Thai-Malaysia border, i.e. Yala, Narathiwas and Songkhla, contained the *pfmdr1* 86Y allele (96.3%) with the mean copy number of 1.2. Malaria cases in the southernmost provinces have significantly increased since 2005, after political unrest in this area. Previously, only a small number of *P. falciparum* isolates collected from this area were genetically characterized. Similar to the present study, Pickard *et al*. identified the *pfmdr1* 86Y allele in all eight *P. falciparum* isolates from Yala, one of the southernmost provinces [13]. This information indicates the different patterns of the *pfmdr1* polymorphisms in *P. falciparum* isolated from different international borders of Thailand. In a few studies of Thai *P. falciparum* isolates, polymorphisms of the *pfmdr1* gene were determined in the parasites mostly collected from either Thai-Myanmar or the Thai-Cambodia borders [10,13,14,17,18]. The recently collected parasites from the Thai-Cambodia border contained the *pfmdr1* 184F allele with a lower copy number compared with the parasites from the Thai-Myanmar border [17,18,29,30]. In contrast, parasites collected from the Thai-Myanmar border usually contained either the 184Y or 184F allele with a higher copy number of the *pfmdr1* gene [17,18,31]. To compare the parasites collected from these three southernmost provinces, the polymorphisms of the *pfmdr1* gene in the parasites from Ranong and Chumphon, provinces along the Thai-Myanmar border, were also determined. Similar to previous studies, most of these parasites, especially those collected from Ranong, had the 184F allele and increased copy number of the *pfmdr1* gene. In contrast to the *pfmdr1* gene, all the study parasites contained the CQ-resistant genotype, the 76T allele.

The different patterns of the *pfmdr1* polymorphisms among the parasites from these international borders of Thailand might be due to the response to a different drug pressure in the past. Since 1995, the combination of ART/MQ has been adopted by the Ministry of Public Health for the treatment of uncomplicated falciparum malaria in Thailand [1,32,33]. In the beginning, this combination was used only in the highly MQ- resistant areas, including the Thai-Myanmar area, Tak and the Thai-Cambodia area, Trat and Chantaburi. In some areas of the Thai-Myanmar border, including Ranong, ART has been added just a few years later. In addition, dosage of ART and MQ might vary among different areas. For example, in 2002, a single dose of 15 mg/kg of MQ was used in the non- or low MQ-resistant areas of Kanchanaburi while in the moderate-MQ-resistant areas of this province, divided doses of 12 mg/kg ART were added. In contrast, in the high MQ-resistant areas such as Mae Sod, Tak province, a combination of 25 mg/kg of MQ and 12 mg/kg of ART was used. The combination of ART/MQ has been used in the malaria clinics of the Office of Disease Prevention and Control in these southernmost provinces since 2004. However, a few hospitals in the area might use other regimens such as a combination of QN/doxycycline. In addition, in Thailand, vivax malaria shares similar endemic areas with falciparum malaria. Thus, CQ, the first-line treatment for vivax malaria could cause a drug pressure and influence the distribution of the *pfmdr1* polymorphisms, especially where vivax malaria was predominant.

The distribution of the *pfmdr1* polymorphisms in each area along the international border of Thailand might be influenced by the parasites from neighbouring countries via human movement. Indeed, approximately half of malaria cases in Thailand have been foreign migrant workers [34]. A few studies of the parasites from the Thai-Cambodia border showed that most parasites collected either from Thailand or Cambodia shared a similar genotype, i e, the *pfmdr1* 184F allele [29,30]. A recent study showed a high level of genetic diversity in the parasites from the Thai-Myanmar and Thai-Cambodia border where cross-border migrations commonly occurred [35]. This study also identified the parasites with the same genotype in patients who were infected in Thailand and Myanmar [35]. In contrast, an identical haplotype was found in all parasites collected from Yala. Similar to this previous study, nearly all parasites collected from three provinces of the Thai-Malaysia border, including Yala, contained the similar *pfmdr1* genotype, the 86Y allele. The distribution of the *pfmdr1* polymorphisms in the parasites from these three southernmost provinces of Thailand was different from those from Pahang, Peninsula Malaysia, containing the *pfmdr1* N86 allele in most isolates [36]. Low variation of the parasite population in this area could be due to recent expansion of the local parasites with fewer introductions of the parasites with other genotypes in the area. Foreign migrant workers in this area were fewer compared to the Thai-Myanmar area, such as Ranong, because of political unrest.

All adapted *P. falciparum* isolates from Yala exhibited a CQ-resistant phenotype. These parasites were QN- and MQ- sensitive. *In vitro* drug susceptibility pattern of the adapted *P. falciparum* isolates in this study was similar to the results of the study by Rungsihirunrat *et al*. [37]. Although the *pfcrt* 76T allele has been proved to be a key determinant for *in vitro* CQ resistance, the polymorphisms of *pfmdr1* gene could modulate the level of CQ resistance [9,10]. Besides, the 86Y allele was also identified as the predictor of CQ treatment failure [38]. It has been shown that parasites with different resistant phenotypes and genotypes exhibited different levels of fitness which might explain the unique parasite structure of *P. falciparum* in the Thai-Malaysia border. The influence of parasites’ fitness was indicated when CQ-sensitive strains were re-emerging in some countries where CQ use was discontinued because of widespread CQ resistance [39,40]. Using allelic replacement technique, insertion of the 7G8, CQ-resistant alleles into CQ-sensitive strain, D10 resulted in the loss of parasites’ fitness [41]. In contrast, a recent study of clinical samples showed that the parasites with the *pfmdr1* 86Y and D1246 alleles had the fitness advantage over others [42]. Moreover, the parasites with the *pfmdr1* 86Y allele could produce a higher number of gametocytes [43] which would gain the advantage in term of transmission.

## Conclusion

A nationwide implementation of a three-day ART instead of a two-day ART in combination with two-day MQ regimen has been started in Thailand since 2008 to overcome a low cure rate in some areas [1]. However, a new fixed dose ACT will inevitably be considered by the Ministry of Public Health in the near future. The present study showed that *P. falciparum* isolated from different areas along the international border of Thailand exhibited different resistant phenotypic and genotypic patterns. This information will be useful for anti-malarial drug policy in Thailand. New candidate drugs should be adopted at least based on their activity against these phenotypic and genotypic parasites in different areas of Thailand.

## Competing interests

The authors declare that they have no competing interests.

## Authors’ contributions

MM and SL conceived of the study, participated in the design and coordination of the study and performed the statistical analysis. SI, PS, SA and ST carried out the field works. NSu performed molecular analysis. NSi carried out the *in vitro* cultivation and sensitivity test. All authors read and approved the final manuscript.
